# Dynamic transcriptome analysis reveals the gene network of gonadal development from the early history life stages in dwarf surfclam *Mulinia lateralis*

**DOI:** 10.1186/s13293-022-00479-3

**Published:** 2022-12-02

**Authors:** Yajuan Li, Liangjie Liu, Lijing Zhang, Huilan Wei, Shaoxuan Wu, Tian Liu, Ya Shu, Yaxin Yang, Zujing Yang, Shi Wang, Zhenmin Bao, Lingling Zhang

**Affiliations:** 1 MOE Key Laboratory of Marine Genetics and Breeding & Sars-Fang Centre, Ocean University of China, 5 Yushan Road, Qingdao, China; 2 Laboratory for Marine Biology and Biotechnology & Laboratory for Marine Fisheries Science and Food Production Processes, Pilot National Laboratory for Marine Science and Technology (Qingdao), Qingdao, China; 3 Key Laboratory of Tropical Aquatic Germplasm of Hainan Province, Sanya Oceanographic Institution, Ocean University of China, Sanya, China

**Keywords:** Gonadal development, Sex differentiation, Gonad formation, Gametogenesis, Gene network, *Mulinia lateralis*

## Abstract

**Background:**

Gonadal development is driven by a complex genetic cascade in vertebrates. However, related information remains limited in molluscs owing to the long generation time and the difficulty in maintaining whole life cycle in the lab. The dwarf surfclam *Mulinia lateralis* is considered an ideal bivalve model due to the short generation time and ease to breed in the lab.

**Results:**

To gain a comprehensive understanding of gonadal development in *M. lateralis*, we conducted a combined morphological and molecular analysis on the gonads of 30 to 60 dpf. Morphological analysis showed that gonad formation and sex differentiation occur at 35 and 40–45 dpf, respectively; then the gonads go through gametogenic cycle. Gene co-expression network analysis on 40 transcriptomes of 35–60 dpf gonads identifies seven gonadal development-related modules, including two gonad-forming modules (M6, M7), three sex-specific modules (M14, M12, M11), and two sexually shared modules (M15, M13). The modules participate in different biological processes, such as cell communication, glycan biosynthesis, cell cycle, and ribosome biogenesis. Several hub transcription factors including *SOX2*, *FOXZ*, *HSFY, FOXL2* and *HES1* are identified. The expression of top hub genes from sex-specific modules suggests molecular sex differentiation (35 dpf) occurs earlier than morphological sex differentiation (40–45 dpf).

**Conclusion:**

This study provides a deep insight into the molecular basis of gonad formation, sex differentiation and gametogenesis in *M. lateralis*, which will contribute to a comprehensive understanding of the reproductive regulation network in molluscs.

**Supplementary Information:**

The online version contains supplementary material available at 10.1186/s13293-022-00479-3.

## Highlights

- *Mulinia lateralis* reaches sexual maturation within 2 months, facilitating comprehensive studies of gonadal development.
- A combined morphological and molecular analysis reveals the timing of gonad formation, sex differentiation and gametogenesis.
- WGCNA on 40 transcriptomes identifies key modules and molecular drivers of clam gonadal development.
- *SOX2*, *FOXZ*, *HSFY*, *FOXL2* and *HES1* are hub transcription factors for gonadal development of *Mulinia lateralis*.

**Supplementary Information:**

The online version contains supplementary material available at 10.1186/s13293-022-00479-3.

## Background

Gonadal development is a complex process consisting of gonad formation, sex differentiation and gametogenesis. This process requires the coordinated expression of a specific set of genes in a strict spatiotemporal manner. In mammals, stem cell-like primordial germ cells migrate to the genital ridge, which gives rise to the bipotential gonad. In this period, *GATA4*, *EMX2*, *LHX9*, and *NR5A1* (also known as *SF1*) are crucial for the formation of genital ridge [1–4], while *SOX2*, *BMP2/4*, *OCT4*, *SOX17*, and *PRDM1/14* are involved in early germ cell specification [5–11]. Sex differentiation occurs under the control of Y-linked transcription factor *SRY*. It activates *SOX9* to drive the testis differentiation [12, 13]. In the absence of *SRY*, female pathways involving *RSPO1*/*WNT4*/*β-catenin* and *FOXL2* are induced, giving rise to the ovaries [14–16]. Finally, gonads undergo proliferation and meiosis to produce mature oocyte or spermatozoa [17]. Many genes are involved in this process, and disruption of the complex mechanisms regulating oogenesis/spermatogenesis may cause infertility.

Mollusca, including bivalves, gastropods, cephalopods, composes the second-largest phylum of animal kingdom after arthropods, with around 200,000 extant species widespread in marine, fresh water and on land [18]. Many molluscs, particularly bivalves such as oysters, scallops, clams and mussels, are an important food source for humans. As economically important aquaculture shellfish, molluscs (17.3 million tons) represented 56.3% of the production of marine and coastal aquaculture in 2018 [19]. They have also gained attention as model species for climate change studies [20]. Research on the gonadal development of molluscs not only helps to understand the molecular mechanism of sex differentiation and gametogenesis, but also assists in unraveling the impact of temperature changes on reproduction.

Molluscs exhibit a diversity of sexual systems, including gonochorism, simultaneous hermaphroditism, and sequential hermaphroditism [21, 22]. Although intensive efforts have been made to elucidate the key genes and pathways regulating gonadal development, most studies focus on germline specification and sexually differentiated gonads. According to previous reports, *NANOS*, *VASA* and *PIWI* are critically important for germ cell development [23–25], and *FOXL2*, *DMRT1L*, *FEM-1-like*, *SOXH*, *VIT* and *TSSK1* play pivotal roles in sex maintenance or gametogenesis [26–29]. Till now, a comprehensive knowledge on the molecular mechanisms of gonadal development including gonad formation, sex determination/differentiation and the transition of gametogenic cycle is still lacking in molluscs. The main obstacle is that most molluscs are difficult to maintain in the lab throughout their lives mainly due to two reasons: (1) many of them have a biphasic life cycle and undergo metamorphosis, which requires special settlement cues that are difficult to obtain; (2) most species have long generation times (generally > 1 year), and cannot be raised under laboratory conditions throughout their lives.

The dwarf surfclam *Mulinia lateralis* is a small marine bivalve native to the Gulf of Mexico, West Indies, and the Atlantic coast [30]. It is considered an ideal mollusc model for genetic research because it has a short generation time (2–3 months) and is easy to maintain and breed in the lab [31]. Since being introduced to China in 2017, *M. lateralis* has been successfully cultured in our lab [32]. Its gonad reaches maturation in 2 months, making it a good material for the study of gonadal development. In present study, we collected the dwarf surfclam from 30 days post-fertilization (dpf) to 60 dpf, and investigated their gonadal development from the morphological and molecular perspectives. It will contribute to a better understanding on the molecular basis of gonad formation, sex differentiation and gametogenesis in *M. lateralis*, and potentially useful for other molluscs.

## Materials and methods

### Sample collection

All experimental dwarf surfclams were cultured in the recirculating aquaculture system in our lab. At least 15 individuals were collected every 5 days from 30 dpf until 60 dpf. Gonads were carefully excised under a stereoscope. Part of them were immediately frozen in liquid nitrogen and then transferred to − 80 °C for RNA extraction, and the rest were fixed in Bouin’s solution overnight at room temperature for histological analysis. Since the gonads cannot be observed until 35 dpf, the molecular studies were initiated at this time point. The testicular and ovarian samples are indicated by the suffix “T” or “O”.

### Histological analysis

After the overnight fixation in Bouin’s solution, the specimens were dehydrated in graded ethanol, cleared in xylene, and embedded in paraffin. Tissue blocks were then cut into 5-µm serial sections on a rotary microtome (Leica, Wetzlar, Germany), and incubated overnight at 37 °C. Finally, the sections were stained with hematoxylin and eosin, and observed under a Nikon’s Eclipse E600 research microscope.

### RNA library construction and sequencing

Total RNA was extracted using the conventional guanidinium isothiocyanate method, followed by DNase I (Takara Bio, Shiga, Japan) digestion to eliminate potential DNA contamination. RNA concentration and purity were determined by Nanovue Plus spectrophotometer (GE Healthcare, New Jersey, USA), and the quality was assessed by agarose gel electrophoresis.

High-quality RNA was used for RNA-Seq library construction following the instructions of the VAHTS Universal V6 RNA-seq Library Prep Kit for Illumina (Vazyme, Nanjing, China). Forty libraries (3–6 biological replicates for each sex at each time point) were subjected to 150 bp paired-end sequencing on the Illumina NovaSeq. All data have been submitted to the NCBI (BioProject ID: PRJNA862073).

### Data preprocessing and normalization

To obtain high-quality (HQ) reads, adapters were removed with Trimmomatic-0.35, and homemade Perl scripts were used to remove reads containing undetermined bases (‘*N*’) or excessive numbers of low-quality positions (> 10 positions with quality scores < 20). The HQ reads were then mapped to the *M. lateralis* genome (unpublished) using STAR [33] with the parameters of ‘STAR -runThreadN 10 -genomeDir fasta -readFilesIn R1.fq R2.fq -sjdbGTFtagExonParentGene gff3 -outFilterMultimapNmax 100,000 -outFileNamePrefix -outReadsUnmapped fasta > STAR_sample.log’. Finally, the raw counts for each gene were obtained by Htseq-count with the parameters of ‘htseq-count -f bam -r name -s reverse -mode = intersection-nonempty -i Parent sample.sorted.bam gff3 > sample.count’, and converted into transcripts per million (TPM) using the RNA-Seq by Expectation Maximization (RSEM).

### Gene co-expression network construction

A signed network was constructed using the R package weighted gene co-expression network analysis (WGCNA) following the procedures described [34]. Briefly, a power of 15 was chosen for the network to exhibit approximate scale-free topology (model fitting index *R*^2^ = 0.75). Subsequently, an adjacency matrix was generated by calculating *Spearman* correlation coefficient between gene pairs. All genes were hierarchically clustered based on dissimilarity measure of topological overlap [35], and the resulting gene dendrogram was used for module detection using the cutreeDynamic method (minModuleSize = 100 and detectCutHeight = 0.99).

### Identification of gonadal development-related modules

To identify gonadal development-related modules, we first identified gonadal development-related genes, which are expected to be differentially expressed among time points or between sex. Here, one-way analysis of variance (ANOVA) was conducted for each gene, and false discovery rate (FDR) was calculated using the *q*-value package to account for multiple tests. Only genes with *q*-value < 0.01 were considered as differentially expressed.

Gonadal development-related modules were determined by overrepresentation analysis of differentially expressed genes (DEGs) using a hypergeometric test, and modules with *q*-value < 0.1 were considered as gonadal development-related modules.

### Functional annotation of genes and modules

Functional annotation of the genes was carried out using BLASTP (*E*-value cutoff 1*e*−5) against the Swiss-Prot database, and the results were imported into Blast2GO. Functional annotation of a module was conducted by GO and KEGG enrichment analyses using GOstats [36], and terms or pathways with adjusted *q*-value < 0.1 were considered as significantly more enriched than random chance in the particular module.

### Hub gene selection and visualization

Hub genes are highly connected genes in a module. A gene’s connection strength to other genes can be indicated by intramodular connectivity *K*_in_. Therefore, top 15% genes with high *K*_in_ were considered as the hub genes for a given module. Co-expression patterns of top 50 hub genes were visualized using the heatmap.

## Results

### Histological analysis of the gonads

**Fig. 1:**
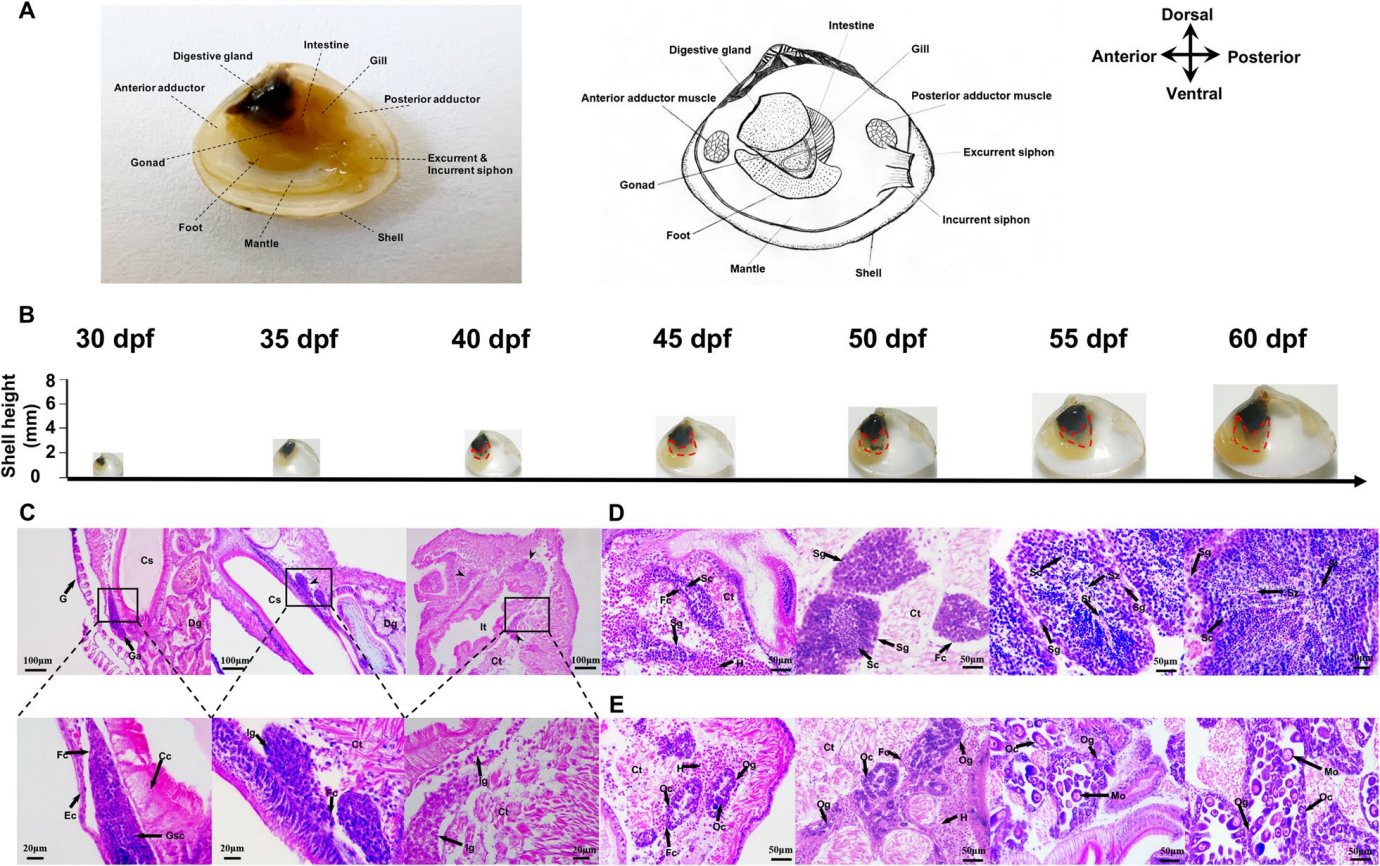
Morphological observation of *Mulinia lateralis* at 30–60 dpf. **A** Photograph (left panel) and schematic diagram (right panel) of the anatomy of *M. lateralis* showing the internal structure (the left shell valve was removed). Anterior toward left and posterior toward right. **B** Anatomy of *M. lateralis* from 30 to 60 dpf showing the shell height. The red dotted lines mark the gonad we collected. **C** Histological observation of the gonads from 30 to 40 dpf. **D** Paraffin sections of the testes from 45 to 60 dpf. **E** Paraffin sections of the ovaries from 45 to 60 dpf. Cc, ciliated cell; Cs, crystalline style; Ct, connective tissue; Dg, digestive gland; Ec, epidermal cell; Fc, follicle cell; G, gill; Ga, gonadal anlage; Gsc, germ stem cell; H, hemocyte; Ig, indistinguishable gonium; IT, intestinal tract; Mo, mature oocytes; Oc, oocyte; Og, oogonia; Sc, spermatocyte; Sg, spermatogonia; St, spermatid; Sz, spermatozoa

The anatomy of *Mulinia lateralis* is shown in Fig. 1A. Considering that *M. lateralis* reaches sexual maturation within 2 months, we examined the gonadal development procedures using juveniles aged 30 (shell height 2.27 ± 0.20 mm) to 60 dpf (shell height 7.70 ± 0.59 mm) (Fig. 1B). As shown in Fig. 1C, germ stem cells (GSCs) are surrounded by a monolayer of follicle cells near the crystalline style at 30 dpf, and the gonads can be observed between digestive gland and foot at 35 dpf; more follicles appear at 40 dpf, each containing sexually indistinguishable gonia. From 45 to 60 dpf, sex can be determined based on the histological analysis (Fig. 1D, E). In the testes, a small number of spermatocytes appear at 45 dpf (shell height 4.92 ± 0.46 mm). Subsequently, the follicles grow bigger, and various types of germ cells are visible at 55 dpf including spermatogonia, spermatocytes, spermatids, and spermatozoa; at 60 dpf, the follicles are predominantly filled with spermatids and spermatozoa (Fig. 1D). In the ovaries, oocytes show up in some follicles at 45 dpf, and more oocytes are observed with the growth of follicles from 50 to 60 dpf. Mature oocytes are visible at 55 and 60 dpf (Fig. 1E).

The histological analysis showed that in *M. lateralis*, gonad formation and sex differentiation occur at 35 and 40–45 dpf, respectively. Subsequently, the gonads go through gametogenic cycle, including a proliferating stage at 45–50 dpf, a growing stage at 50–55 dpf and a mature stage at 55–60 dpf.

### Gonadal transcriptome sequencing and analysis

To better understand the molecular mechanisms of gonadal development in *M. lateralis*, we constructed 40 RNA-Seq libraries using 35–60 dpf gonads (Table 1). A total of 917,474,183 raw reads of 150 bp are obtained, of which 873,981,969 (95.26%) clean reads remained after adapter removal. Among them, 835,574,378 (95.61%) HQ reads are screened by homemade Perl scripts.

**Table 1:** Summary statistics of *M. lateralis* gonad transcriptome sequencing

Sample	Biological replication	Raw reads	Clean reads (%)	HQ reads (%)	NCBI Acc ID
35 dpf	6	131,089,291	125,467,629 (95.71%)	120,504,506 (96.04%)	SAMN29937880-885
40 dpf	6	137,511,710	131,438,793 (95.58%)	125,838,273 (95.74%)	SAMN29937886-891
45 dpf_T	3	67,892,811	64,566,141 (95.10%)	61,911,523 (95.89%)	SAMN29937892-894
45 dpf_O	3	66,575,176	63,469,904 (95.34%)	60,325,179 (95.05%)	SAMN29937906-908
50 dpf_T	3	72,436,212	67,841,216 (93.66%)	64,953,286 (95.74%)	SAMN29937895-897
50 dpf_O	3	69,308,994	65,575,560 (94.61%)	62,900,148 (95.92%)	SAMN29937909-911
55 dpf_T	3	70,153,273	66,537,054 (94.85%)	63,844,072 (95.95%)	SAMN29937898-900
55 dpf_O	3	68,790,439	66,541,194 (96.73%)	63,802,264 (95.88%)	SAMN29937912-914
60 dpf_T	5	118,404,154	113,674,531 (96.01%)	108,186,876 (95.17%)	SAMN29937901-905
60 dpf_O	5	115,312,123	108,869,947 (94.41%)	103,308,251 (94.89%)	SAMN29937915-919
All	40	917,474,183	873,981,969 (95.26%)	835,574,378 (95.61%)	–

**Fig. 2:**
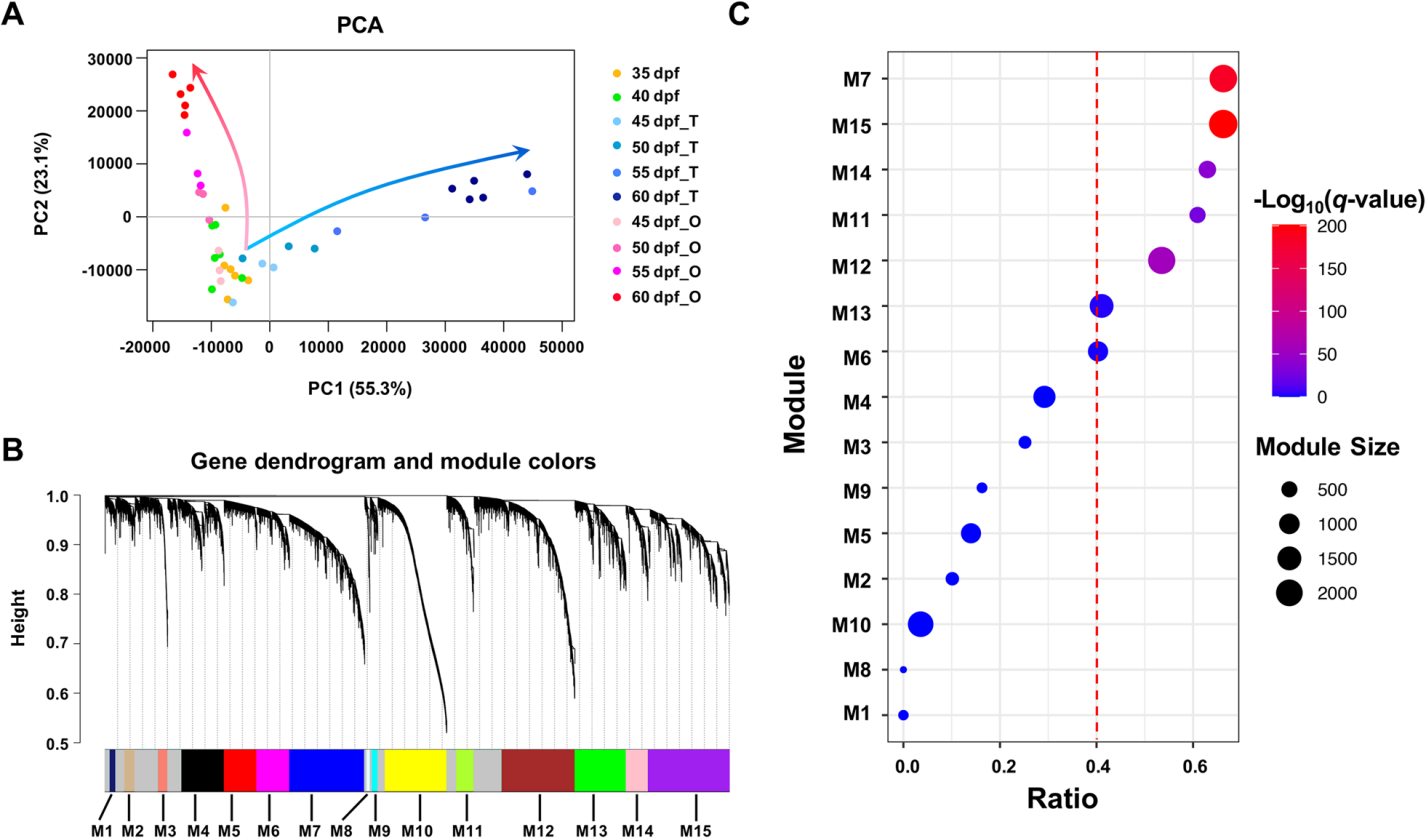
Expression pattern analysis of the gonad samples of *M. lateralis*. **A** The principal component analysis (PCA) score plot of the first two principal components for the 40 gonad samples using 18,074 genes. The red and blue arrows indicate the development of ovary and testis, respectively. **B** Weighted gene co-expression network analysis (WGCNA) of the 40 gonad transcriptomes. Modules of co-expressed genes (M1–M15) are labeled in unique colors, and unassigned genes are labeled in grey. **C** Overrepresentation analysis revealed that seven modules (M7, M15, M14, M11, M12, M13 and M6) were significantly (*q*-value < 0.1) enriched with DEGs

To obtain the relationship between the 40 transcriptomes, we performed a principal component analysis (PCA) on the 18,074 genes expressed in at least one group. According to Fig. 2A, the samples display a trend of differentiation from sexually indistinguishable gonads (35 and 40 dpf) to the mature testes and ovaries. The first principal component (PC1), which explains 55.3% of the variation, discriminates most of the testes from the ovaries and sexually indistinguishable gonads. The gonadal development stages are organized along the second principal component (PC2).

### Network construction and identification of gonadal development-related modules

Gene co-expression network enables identification of modules that represent functional categories [37–39]. Here, 18,074 genes from the 40 gonadal transcriptomes were used for network construction, resulting in 15 modules (M1 ~ M15) with module size ranging from 103 to 2350 genes (Fig. 2B).

A total of 6986 DEGs (*q*-value < 0.01) are identified by differential expression analysis, of which 6768 (~ 97%) are included in the network construction. Overrepresentation analysis revealed that seven modules (*q*-value < 0.1) including M15 (*q*-value = 6.53*e*−202), M7 (*q*-value = 2.54*e*−185), M12 (*q*-value = 8.84*e*−57), M14 (*q*-value = 7.07*e*−40), M11 (*q*-value = 1.42*e*−27), M13 (*q*-value = 3.95*e*−3) and M6 (*q*-value = 0.07) are significantly enriched with DEGs (Fig. 2C). The proportion of DEGs is greater than 40% in each of these modules.

**Fig. 3:**
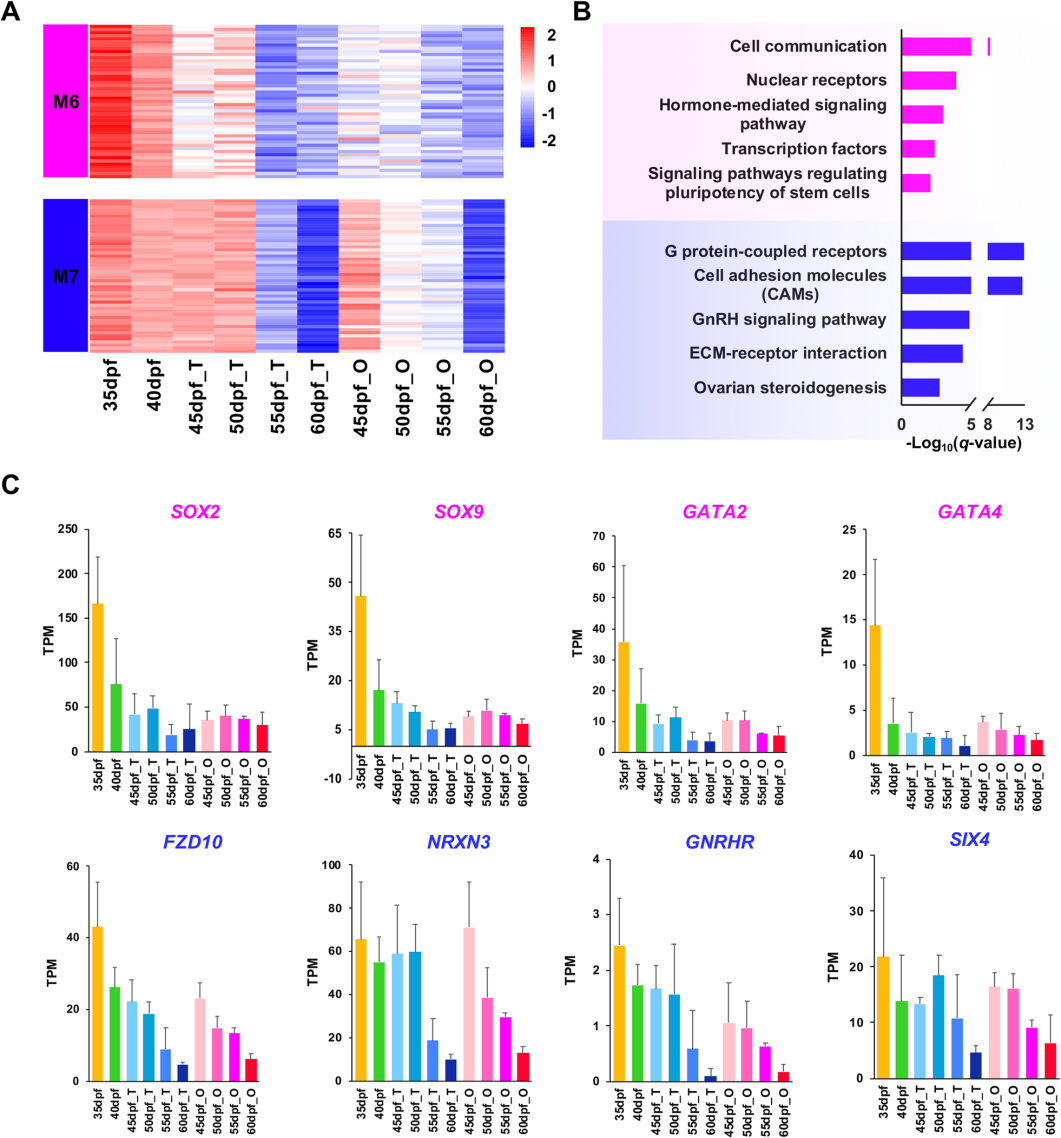
Gene expression pattern and functional enrichment of the two gonad-forming modules. **A** Heatmap visualization of top 50 hub genes in modules M6 and M7. **B** GO and KEGG enrichment analysis of genes in M6 and M7. **C** The expression profiles of several representative genes from the two modules in each time point. The gene names are highlighted with the corresponding module color

**Fig. 4:**
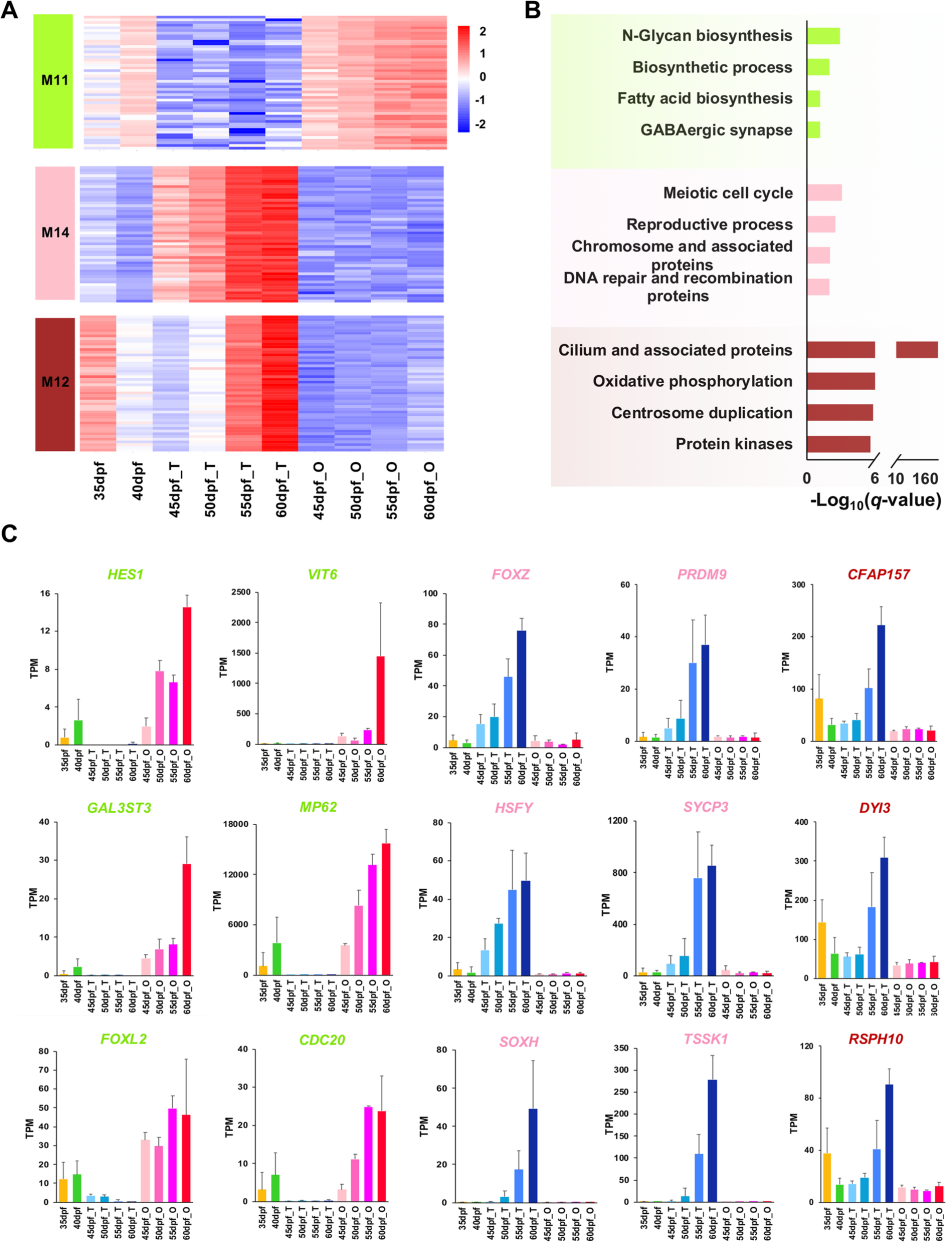
Gene expression pattern and functional enrichment of the three sex-specific modules. **A** Heatmap visualization of top 50 hub genes in modules M11 (ovary biased), M14 and M12 (testis biased). **B** GO and KEGG enrichment analysis of genes in the three modules. **C** The expression profiles of several representative genes from the three modules. The gene names are highlighted with the corresponding module color

**Fig. 5:**
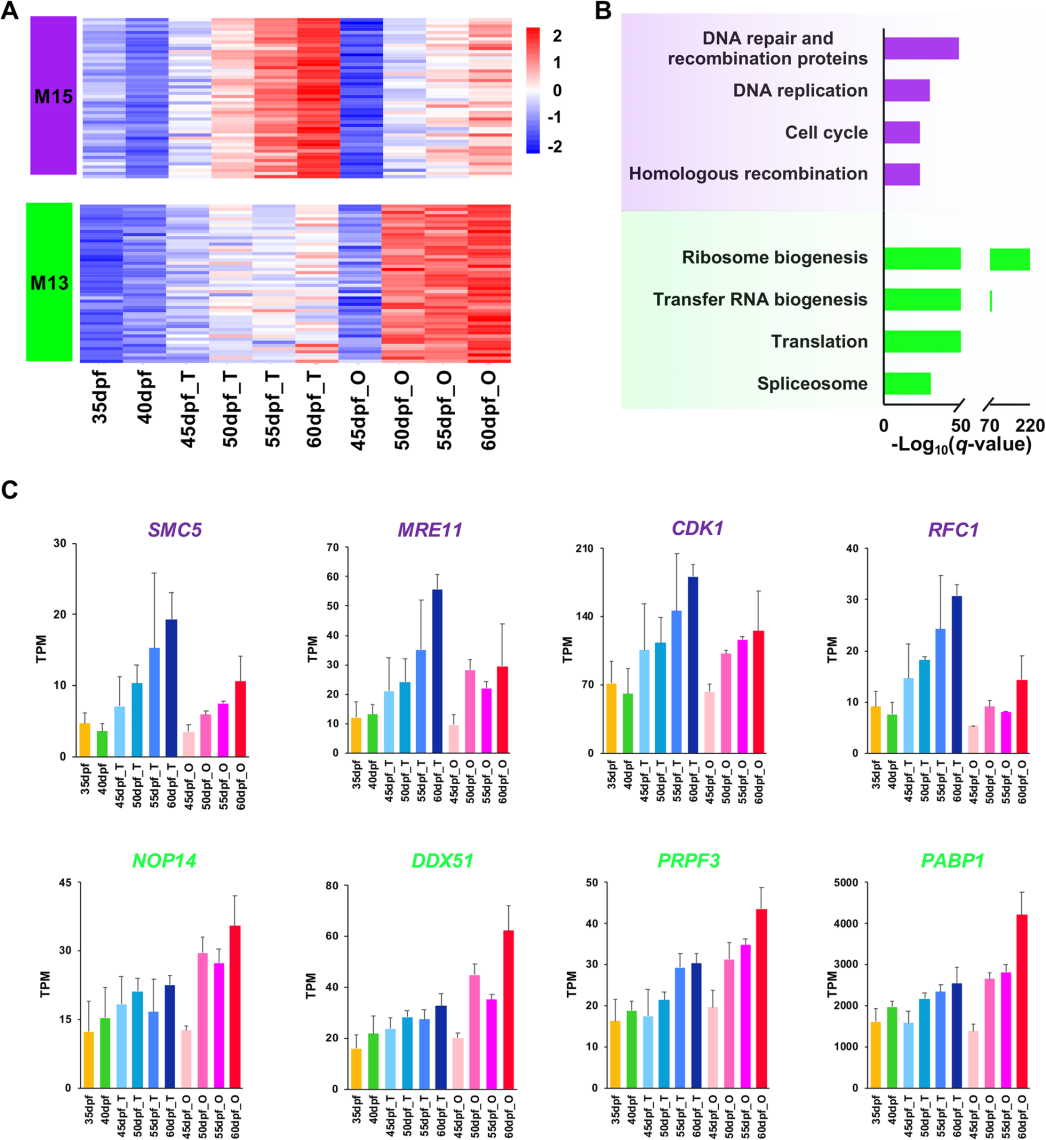
Gene expression pattern and functional enrichment of the two sexually shared modules. **A** Heatmap visualization of top 50 hub genes in modules M15 and M13. **B** GO and KEGG enrichment analysis of genes in M15 and M13. **C** The expression profiles of several representative genes from the two modules. The gene names are highlighted with the corresponding module color

To determine to which gonadal development process the DEGs enriched modules are related, we investigated the gene expression profiles of top 50 hub genes in these modules (Additional file 1: Table S1). Results showed that the top 50 hub genes of M6 and M7 modules are highly expressed during early stage at 35 and 40 dpf, and for M7, the expression continues to the early differentiated testes and ovaries (Fig. 3A). Hub genes of M14, M12 and M11 display sex-specific expression patterns, with M14 and M12 being testis specific, and M11 being ovary specific (Fig. 4A). Hub genes of M15 and M13 are expressed at all stages of gonads, but the expression level is higher in the mature gonads than in the early ones (Fig. 5A). According to the expression patterns, these modules are considered as gonad-forming modules (M6, M7), sex-specific modules (M14, M12, M11), and sexually shared modules (M15, M13), respectively.

### Functional annotation and hub genes of gonad-forming modules

To further understand the biological roles of these gonadal development-related modules, GO and KEGG enrichment analyses were performed. The gonad-forming module M6 is enriched with genes involved in cell communication (*q*-value = 5.03*e*−9), nuclear receptors (*q*-value = 1.11*e*−4), transcription factors (*q*-value = 4.22*e*−3) and signaling pathways regulating pluripotency of stem cells (*q*-value = 8.10*e*−3) (Fig. 3B). Transcription factor *SOX2* has the highest intramodular connectivity, suggesting it may be a key driver for early gonadal development. The other genes such as transcription factors *SOX9*, *GATA2* (GATA-binding factor 2), *OVO* (transcriptional regulator ovo), nuclear receptors *NR2F1* (nuclear receptor subfamily 2 group F member 1)*, NR1D3* (nuclear receptor subfamily 1 group D member 3) and *ER* (estrogen receptor), cell communication-related genes *WNT7B* (protein Wnt-7b precursor) and *HTR4* (5-hydroxytryptamine receptor 4) are among the top 15% hub genes.

The other gonad-forming module M7 is enriched for G protein-coupled receptors (*q*-value = 1.50*e*−13), cell adhesion molecules (*q*-value = 2.41*e*−13), GnRH signaling pathway (*q*-value = 1.28*e*−5) and ECM-receptor interaction (*q*-value = 4.02*e*−5) (Fig. 3B). The top 15% hub genes include G protein-coupled receptors *5-HTR1* (5-hydroxytryptamine receptor 1), *FZD10* (Frizzled-10) and *SSTR2* (Somatostatin receptor type 2), cell adhesion molecules *NRXN3* (Neurexin-3) and *NLGN4Y* (Neuroligin-4, Y-linked), *Rut* (Ca(2+)/calmodulin-responsive adenylate cyclase) and *GNRHR* (gonadotropin-releasing hormone receptor) from GnRH signaling pathway, and several collagens (*COL4A5*, *COL6A6* and *COL1A2*).

Figure 3C shows the dynamic expression profiles of some representative genes of M6 and M7 during gonadal development. Except for the above-mentioned hub genes, we found some previously reported early gonadal development-related genes, such as *GATA4* (GATA-binding factor 4) and *SIX4* (homeobox protein SIX4), are also in module M6 and M7, and display similar expression pattern with the hub genes.

### Functional annotation and hub genes of sex-specific modules

The ovary-specific module M11 is enriched with genes participating in N-glycan biosynthesis (*q*-value = 1.25*e*−3), biosynthetic process (*q*-value = 1.04*e*−2), and fatty acid biosynthesis (*q*-value = 6.71*e*−2) (Fig. 4B). Top 15% hub genes including *HES1* (transcription factor HES-1), *GAL3ST3* (Galactose-3-*O*-sulfotransferase 3), *FOXL2* (forkhead box protein L2) from biosynthetic process, *FASN* (Fatty acid synthase) from fatty acid biosynthesis, *RPN1* (26S proteasome regulatory subunit RPN1) and *DDOST* (dolichyl-diphosphooligosaccharide–protein glycosyltransferase 48 kDa subunit) from N-glycan biosynthesis are specifically expressed in the ovaries. Similar expression patterns are also detected for *VIT6* (Vitellogenin-6), a female-specific glycoprotein, as well as some cell cycle genes, such as *MP62* (mitotic apparatus protein p62) and *CDC20* (cell division cycle protein 20) (Fig. 4C)*.*

The testis-specific modules are enriched with spermatogenesis-related categories. Specifically, M14 is overrepresented with genes involved in meiotic cell cycle (*q*-value = 7.63*e*−4) and reproductive process (*q*-value = 2.95*e*−3) (Fig. 4B). Representative genes such as transcription factors *FOXZ* (Forkhead box protein Z), *HSFY* (heat shock transcription factor, Y-linked) and *SOXH* (transcription factor SOXH), chromosome and associated proteins including tubulins, *PRDM9* (histone-lysine *N*-methyltransferase PRDM9) and *SYCP3* (Synaptonemal complex protein 3), DNA repair and recombination proteins including *HFM1* (Probable ATP-dependent DNA helicase HFM1) and *MSH4* (MutS protein homolog 4), display similar expression patterns, with specific expression in the testes and especially high in later stages. Spermiogenesis related genes, such as testis-specific serine/threonine-protein kinase family genes (*TSSK1*, *TSSK4*) are also present in module M14 and highly expressed in the testes (55 and 60 dpf) (Fig. 4C).

The other testis-specific module M12 is enriched for cilium and associated proteins (*q*-value = 1.42*e*−225), oxidative phosphorylation (*q*-value = 2.16*e*−7), and protein kinases (*q*-value = 2.46*e*−6) (Fig. 4B). Representative hub genes from these terms include cilia- and flagella-associated proteins, axonemal dyneins and radial spoke head proteins, which are highly expressed in 60 dpf testes and moderately in 55 dpf testes and 35 dpf gonads (Fig. 4C).

### Functional annotation and hub genes of sexually shared modules

The two sexually shared modules M15 and M13 are enriched with biological processes commonly exist in spermatogenesis and oogenesis. M15 is enriched with genes participating in cell cycle (*q*-value = 3.60*e*−24), DNA replication (*q*-value = 1.05*e*−30) and repair (*q*-value = 8.44*e*−50) (Fig. 5B). Representative hub genes such as *SMC5* (structural maintenance of chromosomes protein 5), *MRE11* (double-strand break repair protein MRE11), *CDK1* (cyclin-dependent kinase 1) and *RFC1* (replication factor C subunit 1) are primarily expressed in the late stages of testes and ovaries, with the peak in 60 dpf testes (Fig. 5C).

Module M13 is enriched for ribosome biogenesis (*q*-value = 4.75*e*−219), translation (*q*-value = 8.32*e*−73), and spliceosome (*q*-value = 3.94*e*−31) (Fig. 5B). Hub genes such as *NOP14* (nucleolar protein 14), *DDX51* (ATP-dependent RNA helicase DDX51), *DDX27* (Probable ATP-dependent RNA helicase DDX27), *DDX54* (ATP-dependent RNA helicase DDX54), *EIF3C* (eukaryotic translation initiation factor 3 subunit C), *PRPF3* (U4/U6 small nuclear ribonucleoprotein Prp3) and *PABP1* (polyadenylate-binding protein 1) are highly expressed in the late stages of testes and ovaries, with the peak in 60 dpf ovaries (Fig. 5C).

### Timing of molecular sex differentiation

**Fig. 6:**
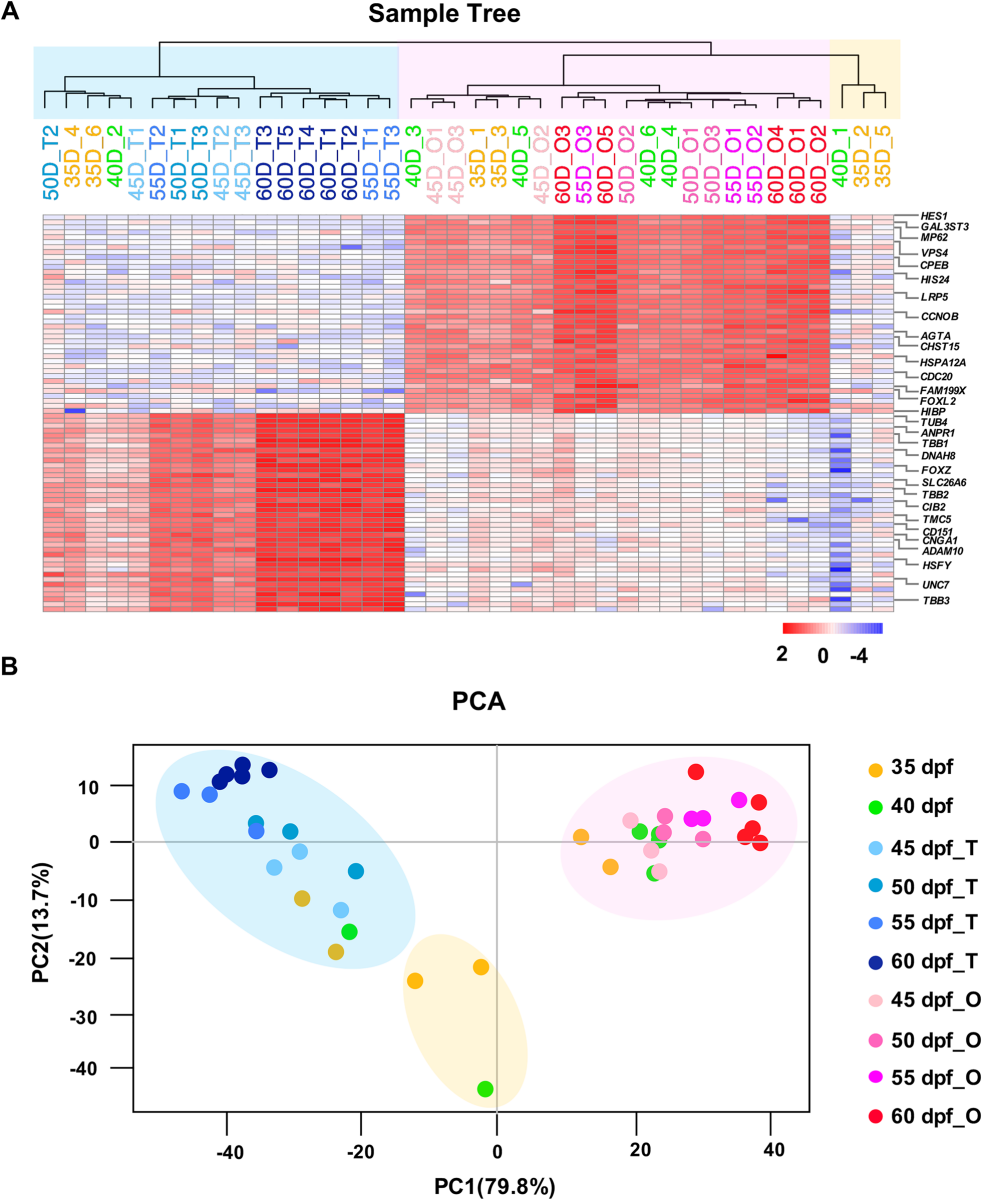
Determination of the timing of molecular sex differentiation. **A** Hierarchical clustering on all gonad samples based on the expression patterns of 80 sex differentiation-related genes from modules M11 and M14. **B** The PCA score plot of the first two principal components for the 40 gonad samples using the 80 sex differentiation-related genes. The ovaries, testes and undifferentiated gonads are shown with light pink, blue and yellow backgrounds, respectively

To determine the timing of molecular sex differentiation in *M. lateralis*, we selected sex-specific genes for hierarchical clustering analysis and PCA on the 40 samples. Considering that M11 and M14 are sex-specific modules with genes consistently highly expressed from 45–60 dpf, we chose top 40 hub genes that show differential expression (*p*-value < 0.05) between genders at 45 dpf from each module. As shown in Fig. 6, similar results were obtained for hierarchical clustering and PCA analyses, showing that the 40 samples can be clustered into three groups (ovaries, testes and undifferentiated gonads). The genders of dwarf surfclams at 45–60 dpf are easily distinguished, consistent with the morphological results. In addition, the sex of most of the sexually indistinguishable gonads at 35 and 40 dpf can also be determined, with the ratio of 67% (4/6) and 83% (5/6), respectively.

### Summary of the developmental process of *M. lateralis* gonads

**Fig. 7:**
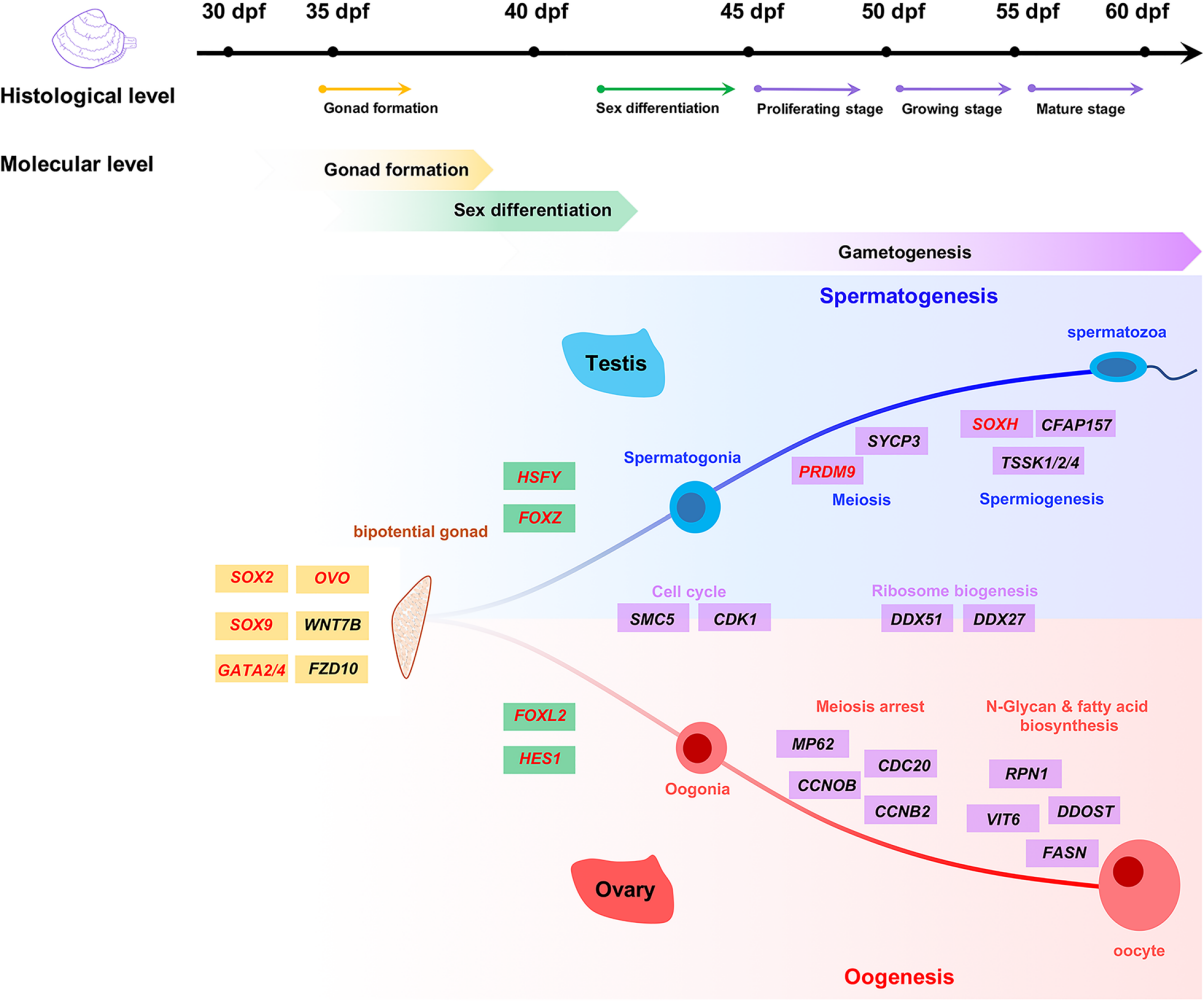
Schematic diagram of the gonadal development of *M. lateralis* based on the histological and molecular analyses. The diagram shows the timing of gonad formation (light yellow), sex differentiation (light green), and different stages of gametogenesis (light purple) at both histological and molecular levels. The representative genes for these processes are shown in rectangles with the corresponding background colors. The key transcription factors are marked in red

Based on the above results, the gonadal developmental process of *M. lateralis* is summarized at histological and molecular levels (Fig. 7). Gonad formation occurs as early as 35 dpf, in which some cell communication-related genes, G protein-coupled receptors, and transcription factors are involved, such as *SOX2*, *SOX9*, *GATA2/4*, *OVO*, *WNT7B* and *FZD10*. At 35–40 dpf, molecular sex differentiation occurs, with the elevation of *HSFY* and *FOXZ* in the testis, and the induction of *FOXL2* and *HES1* in the ovary. At 45 dpf, sex can be distinguished based on histological observation, and the gonads start to go through gametogenic cycle. Cell cycle and ribosome biogenesis-related genes are highly expressed in the ovary and testis during the gametogenesis, such as *SMC5*, *CDK1*, *DDX51* and *DDX27*. But chromosome and cilium-related genes such as *PRDM9*, *SYCP3*, *SOXH* and *CFAP157* are specifically expressed in the testis, participating in the spermatogenesis. In the ovary, N-glycan and fatty acid biosynthesis related genes such as *RPN1*, *DDOST*, *VIT6* and *FASN* are highly expressed, as well as some meiosis arrest-related genes like *MP62*, *CDC20*, *CCNO-B* and *CCNB2*, which could play vital roles in oocyte development.

## Discussion

Gonadal development involves various biological processes, such as gonad formation, sex differentiation, and gametogenesis. Comprehensive studies of gonadal development require continuous sampling until sexual maturation. For example, in *Patinopecten yessoensis* [40], *Argopecten irradians* [41] and *Crassostrea sikamea* [42], individuals aged 5–13, 2–8 and 1–15 months old were tracked to illustrate sex differentiation and gametogenesis, respectively. Long generation time (usually 1–2 years) [43] limits the studies of gonadal development for most molluscs. In contrast, *Mulinia lateralis* has been reported to reach sexual maturation within 2–3 months [31, 43], which facilitates comprehensive studies of gonadal development. Our study confirms that *M. lateralis* become mature at 60 dpf. Most importantly, based on the histological analysis of gonads from 30–60 dpf every 5 days, we determined the timing of gonad formation, sex differentiation and gametogenesis. It provides a valuable resource for studies of the molecular mechanisms of gonadal development, and will facilitate more precise regulation of *M. lateralis* reproduction in the lab.

Transcriptome analysis has been widely used to elucidate the molecular mechanisms underlying various traits. However, it often produces many DEGs, and the key drivers are difficult to identify. WGCNA represents a transition from gene-centric to network or module-centric approaches. It enables researchers to identify important modules and central players within modules, and has proven its power in many organisms including molluscs [39, 44–47]. In this study, a gene co-expression network was constructed based on 40 transcriptomes covering the entire gonadal development process of *M. lateralis,* allowing a comprehensive understanding of the molecular changes and key drivers for gonad formation, sex differentiation and gametogenesis.

Among the 7 gonadal development-related modules, the three sex-specific modules are enriched with GO terms or KEGG pathways that have been reported in previous studies [26, 28, 48–51]. Specifically, enrichment of N-glycan biosynthesis, fatty acid biosynthesis and GABAergic synapse in the ovary is also found in *P. yessoensis* [26, 48], *Pinctada margaritifera* [28] and *Chlamys farreri* [49], suggesting these biological processes may be critical for ovary development in various mollusc species. Cell cycle, sexual reproduction and oxidative phosphorylation have also been reported to be overrepresented in the testis-biased genes in *P. yessoensis* [26], *Nodipecten subnodosus* [50] and *Crassostrea gigas* [51]. According to the functional annotation and key genes, the two testis-specific modules of *M. lateralis* correspond to the turquoise and green modules of *P. yessoensis* [48], suggesting the biological processes involved in the two modules are widely present in different molluscs.

In addition to sex-specific modules, we identified two sexually shared modules (M15 and M13). These modules participate in general biological processes such as DNA replication, repair, and translation, which occur in the testes and ovaries. Although similar modules or functional categories have been reported in other molluscs, they are generally considered to be sex biased [48, 51]. Specifically, DNA replication and repair are enriched in testis-biased genes, likely because there are more cells going through cell cycles in the testes than ovaries. Ribosome biogenesis and translation are enriched in ovary-biased genes, possibly because these processes occur more frequently in the oocytes than the male gametes. The sexual-biased expression patterns may cause these biological processes to be overlooked in the other sex and should receive more attention in future studies.

As is discussed above, previous studies of mollusc gonad development primarily focused on the gametogenic cycle and sexual differentiation [52–55], whereas studies on gonad formation are lacking. Since consecutive gonadal samples can be obtained for *M. lateralis* in the early stages, they can be used to construct gene network and identify gonad-forming modules. The two modules we identified consist of genes that show highest expression in 35 dpf gonads, which is quite different from the expression patterns of sex-specific or sexually shared modules. Functional annotation suggests involvement of cell communication, hormone-mediated signaling pathway, G protein-coupled receptors and ECM–receptor interaction in gonad formation. Similarly, several wnt ligands and their receptors Fzs from Wnt signaling pathway have been demonstrated to play a vital role in the stemness regulation of mammalian spermatogonial stem cells [56]. In *Xenopus laevis*, signaling molecules (e.g., *WNT7B*), extracellular matrix molecules (e.g., *collagens*) and transcription factors (e.g., *LHX8*) also play important roles at the initial stage of gonadogenesis [57]. These studies suggest a conserved role of wnt signaling pathway and ECM–receptor interaction in gonadogenesis in different animals.

Transcription factors are well known to play critical roles in diverse biological processes, including gonadogenesis [4, 58] and sexual development [16, 59]. Our study reveals that transcription factors *SOX2*, *SOX9*, *GATA2* and *OVO* are important for gonad formation, *FOXL2* and *HES1* for ovary development, and *HSFY*, *FOXZ* and *SOXH* for testis development in *M. lateralis*. Among them, *SOX2*, *FOXL2*, *FOXZ* and *SOXH* have been reported to be vital for gonadal development in other molluscs [40, 41, 60–64]. Studies on *FOXL2* have demonstrated its central role in sex differentiation and ovary maintenance in several mollusc species [40, 41, 60–62]. The other genes (*SOX2*, *SOXH* and *FOXZ*) are reported to be essential for spermatogenesis and testis development in scallops and oysters [60, 63, 64]. Except for the protostome-specific *FOXZ*, the remaining three genes seem to play conserved roles in vertebrates. Specifically, *FOXL2* also exerts critical roles in sex determination [14], sex differentiation [65], and gametogenesis [66]. *SOX2* is essential for PGC (primordial germ cell) proliferation [5], and *SOXH* is associated with testis development and required for male fertility [67, 68]. The remaining transcription factors are rarely studied in molluscs, but some of them have been demonstrated to be involved in the sexual development of other animals. For example, the roles of *SOX9* and *HSFY* in testis development have been well studied in mammals. *SOX9* is the target of *SRY*, which mediates testis determination and differentiation [69, 70]. *HSFY* is a Y-linked gene in human whose deletion results in male infertility, and is also involved in the spermatogenesis in other animals [71–74]. *OVO*, *HES1* and *GATA2* are also reported to play essential roles in ovary development in various organisms. For example, *OVO* is important for female germline survival and oogenesis in insects [75, 76]. *HES1* is the major target genes of NOTCH signaling, which regulates ovarian somatic cell development and is necessary for oocyte maturation in mammals [17, 77]. *GATA2* is transiently expressed in the ovarian germ cell lineage during mouse gonadogenesis, and the signal location coincides with germ cell marker *OCT4* [78]. Some of the important transcription factors (e.g., *FOXL2*, *SOX9* and S*OXH*) we found in *M. lateralis* are also identified as key regulators of sex development in mouse [79], tilapia [80] and sea bass [81] by WGCNA, suggesting the drivers of gonadal development may be conserved across animal phyla.

The onset of sex differentiation can be determined by the morphological or molecular method [80, 82]. In this study, we found molecular sex differentiation occurs as early as 35 dpf, while morphological sex differentiation occurs at 40–45 dpf in *M. lateralis*. It suggests sex differentiation occurs earlier at the molecular level than at the morphological level, similar to the findings in scallops *P. yessoensis* [40] and *A. irradians* [41]. But unlike scallops in which *FOXL2* and *DMRT1L* are the key sex differentiation genes, we found that only *FOXL2* shows sexual dimorphic expression in *M. lateralis*. This confirms that the role of *FOXL2* in the ovary development is likely to be deeply conserved in molluscs. DM domain genes play central roles in sex differentiation in many metazoans, including fish, birds, nematodes and arthropods [83]. However, it may not be involved in sex differentiation of *M. lateralis*. We speculate that other transcription factors, such as *FOXZ* and *HSFY*, may replace *DMRT1L* to promote testis development.

## Perspectives and significance

Mollusca composes the second-largest phylum of animal kingdom after arthropods, and is an important food source for humans. Our study on the gonadal development of dwarf surfclam *M. lateralis*, which is considered an ideal bivalve model for genetic research, provides detailed information on the gene network of gonad formation, sex differentiation and gametogenesis. It will facilitate reproductive control in molluscs. The hub transcription factors we identified could be key drivers in mollusc gonadogenesis, sex differentiation or gamete development. In the future, comprehensive studies on the expression, regulation and function of these transcription factors are needed to elucidate the molecular mechanism of mollusc sexual development and to reveal its consistency/differences with other animal phyla.

## Supplementary Information

**Additional file 1: Table S1.** Detailed information on WGCNA, differential expression analysis and gene annotation for the seven gonadal development-related modules.

## Data Availability

All data generated or analyzed during this study are included in this published article and its additional information files.

## Abbreviations

dpf: Day post-fertilization
TPM: Transcripts per million
ANOVA: One-way analysis
FDR: False discovery rate
DEGs: Differentially expressed genes
GSCs: Germ stem cells
PCA: Principal component analysis
*GATA2*: GATA-binding factor 2
*OVO*: Transcriptional regulator ovo
*NR2F1*: Nuclear receptor subfamily 2 group F member 1
*NR1D3*: Nuclear receptor subfamily 1 group D member 3
*ER*: Estrogen receptor
*WNT7B*: Protein Wnt-7b precursor
*HTR4*: 5-Hydroxytryptamine receptor 4
*5-HTR1*: 5-Hydroxytryptamine receptor 1
*FZD10*: Frizzled-10
*SSTR2*: Somatostatin receptor type 2
*NRXN3*: Neurexin-3
*NLGN4Y*: Neuroligin-4, Y-linked
*Rut*: Ca(2+)/calmodulin-responsive adenylate cyclase
*GNRHR*: Gonadotropin-releasing hormone receptor
*GATA4*: GATA-binding factor 4
*SIX4*: Homeobox protein SIX4
*HES1*: Transcription factor HES-1
*GAL3ST3*: Galactose-3-*O*-sulfotransferase 3
*FOXL2*: Forkhead box protein L2
*FASN*: Fatty acid synthase
*RPN1*: 26S proteasome regulatory subunit RPN1
*DDOST*: Dolichyl-diphosphooligosaccharide–protein glycosyltransferase 48 kDa subunit
*VIT6*: Vitellogenin-6
*MP62*: Mitotic apparatus protein p62
*CDC20*: Cell division cycle protein 20
*FOXZ*: Forkhead box protein Z
*HSFY*: Heat shock transcription factor, Y-linked
*SOXH*: Transcription factor SOXH
*PRDM9*: Histone-lysine *N*-methyltransferase PRDM9
*SYCP3*: Synaptonemal complex protein 3
*HFM1*: Probable ATP-dependent DNA helicase HFM1
*MSH4*: MutS protein homolog 4
*TSSK1*/4: Testis-specific serine/threonine-protein kinase family genes *¼*
*SMC5*: Structural maintenance of chromosomes protein 5
*MRE11*: Double-strand break repair protein MRE11
*CDK1*: Cyclin-dependent kinase 1
*RFC1*: Replication factor C subunit 1
*NOP14*: Nucleolar protein 14
*DDX51*: ATP-dependent RNA helicase DDX51
*DDX27*: Probable ATP-dependent RNA helicase DDX27
*DDX54*: ATP-dependent RNA helicase DDX54
*EIF3C*: Eukaryotic translation initiation factor 3 subunit C
*PRPF3*: U4/U6 small nuclear ribonucleoprotein Prp3
*PABP1*: Polyadenylate-binding protein 1
